# The association between mental health nursing and hospital admissions for people with serious mental illness: a protocol for a systematic review

**DOI:** 10.1186/s13643-017-0658-5

**Published:** 2018-01-09

**Authors:** Matthew J. Leach, Martin Jones, Dan Bressington, Fiona Nolan, Adrian Jones, Kuda Muyambi, Marianne Gillam, Richard Gray

**Affiliations:** 1 Department of Rural Health, University of South Australia, North Terrace, Adelaide, South Australia 5000 Australia; 2 Department of Rural Health, University of South Australia, 111 Nicholson Avenue, Whyalla Norrie, South Australia 5608 Australia; 3 Hong Kong Polytechnic University, Hung Hom, Kowloon, Hong Kong, Special Administrative Region of China; 4 School of Health and Human Science, University of Essex, Wivenhoe Park, Colchester, Essex, CO4 3SQ UK; 5 Betsi Cadwaladr University Health Board, Wrexham Maelor Hospital, LL167TD, Wrexham, UK; 6 La Trobe University, Bundoora, Victoria 3086 Australia

**Keywords:** Hospital admission, Hospitalisation, Mental health nursing, Mental illness, Psychiatric nursing, Systematic review

## Abstract

**Background:**

Relapse in individuals with severe mental illness (SMI) is a frequent occurrence and can add considerably to the burden of disease. As such, relapse prevention is an essential therapeutic outcome for people with SMI. Mental health nurses (MHNs) are well placed to support individuals with SMI and to prevent relapse; notwithstanding, there has been no synthesis of the evidence to date to determine whether MHNs prevent relapse in this population.

**Methods:**

Electronic databases will be systemically searched for observational studies and clinical trials that report the association between mental health nursing and the hospitalisation of persons living with an SMI. The search will be supplemented by reference checking and a search of the grey literature. The primary outcome of interest will be hospital admission rate. Screening of articles, data extraction and critical appraisal will be undertaken by two reviewers, independently, with a third reviewer consulted should disagreement occur between reviewers. The quality of studies will be assessed using the Risk Of Bias In Non-randomised Studies - of Interventions (ROBINS-I) tool and the Cochrane Collaboration risk of bias tool. Depending on the number of studies and level of heterogeneity, the evidence may be synthesised using meta-analysis or narrative synthesis.

**Discussion:**

This review will explore for the first time the clinical potential of mental health nursing in preventing relapse in persons with SMI. The findings of this review will serve to inform future research and education in this area. The evidence may also help inform future policy, including decisions regarding future mental health workforce development and planning.

**Systematic review registration:**

PROSPERO CRD42017058694.

## Background

Mental disorders represent a highly prevalent and burdensome group of diagnosable health conditions. Across the globe, close to one in three people (29%) have experienced a common mental disorder some point in their lifetime [1]. The burden of these disorders is considerable, accounting for 183.9 million disability-adjusted life years (DALYs) or 7.4% of all DALYs worldwide. More than 14% of the DALYs caused by mental and substance use disorders are in people with severe mental illness [2].

Serious mental illness (SMI) is a diagnosis of any non-organic psychosis, with a duration of treatment of 2 years or more, and evidence of dysfunction (as measured by the Global Assessment of Functioning scale) [3]. Conditions captured within SMI include schizophrenia, schizophrenia-like disorders, bipolar disorder and depression with psychotic features [4]. People with SMI are a particularly vulnerable group, with reports of increased risk of criminal victimisation (2.3 to 140-fold increased risk) [5], hospitalisation [6], falls [6], emergency department presentations (2.9-fold increased risk) [7] and mortality (2.2-fold increased risk) [4] when compared with the general population.

Relapse (i.e. the worsening of psychopathological symptoms or re-hospitalisation within 12 months of discharge from hospital) [8] in people with SMI is a frequent occurrence. Approximately 20% of individuals with schizophrenia will relapse [9]; for people with bipolar disorder, the rate of relapse may be as high as 40% [10]. A high proportion of relapses (i.e. 75% of cases) in people with schizophrenia result in admission to hospital [8]; this adds considerably to health care expenditure and further burdens health care resources [9]. Relapse (and subsequent hospitalisation) is also distressing for the person with SMI and their family [11]. Furthermore, a relapse experience in persons living with serious mental illness increases the likelihood of further relapse [9]. It is no surprise then that relapse prevention is a key therapeutic aim of SMI [12].

Case management is a comprehensive approach to managing serious mental illness, with evidence suggesting that intensive case management may be effective in reducing hospitalisation in people with SMI [13]. Mental health nurses (MHNs) account for a considerable proportion of case managers; they also represent the greatest percentage of the mental health workforce [14]. Consequently, MHNs are likely to have the largest face-to-face time with people living with SMI than any other discipline [15]. As such, MHNs are well placed in their role to support people with SMI and to prevent relapse.

Despite the broad implications of relapse in people with SMI and the assumed role of MHNs in mitigating admissions to hospital in this population, there has been no synthesis of the evidence to ascertain whether MHNs can prevent relapse in people with SMI. This systematic review addresses this knowledge gap by investigating the relationship between MHN exposure and hospitalisation-related outcomes in individuals with SMI.

## Methods

### Research objectives

The aim of this review will be to systematically review, appraise and synthesise the evidence from studies reporting the association between mental health nursing and the hospitalisation of persons living with SMI.

### Eligibility criteria

#### Study designs

The review will consider published and unpublished observational studies (including cross-sectional, cohort and case-control studies) and clinical trials (including randomised controlled trials, non-randomised controlled trials and comparative studies) that address the research objective. Excluded will be qualitative investigations and literature reviews.

#### Participants

Participants will be limited to community-dwelling people diagnosed with SMI, which includes schizophrenia, schizophrenia-like disorders, bipolar disorder and depression with psychotic features [4]. Excluded will be persons living in secure settings and hospital inpatients.

#### Interventions

Studies that examine the effect of care provided by mental health nurses only will be eligible for inclusion. For the purpose of this review, a mental health or psychiatric nurse is someone who has completed a formal qualification in mental health/psychiatric nursing and has been credentialed, licensed or registered to practice in that capacity (e.g. registered psychiatric nurse). Studies examining the effect of team-based models of care, or specific MHN-administered clinical interventions (e.g. cognitive behavioural therapy, adherence therapy), will be excluded.

#### Comparators

The review will consider any comparison, including other health service providers (e.g. psychologists, psychiatrists), team-based models of care, non-MHN exposure and services provided by non-government organisations.

### Outcomes

The review focuses on parameters related to hospitalisation and safety, as outlined below.

#### Primary outcomes

- Hospital admission rate (e.g. 30-day admission rate)

#### Secondary outcomes

- Hospital length of stay (e.g. mean number of hospital bed days)
- Emergency department (ED) presentations (e.g. mean number of ED presentations, proportion of ED presentations resulting in hospital admission)
- Home treatment/crisis team presentations (e.g. team referrals and admissions)
- Number of days treated by home treatment/crisis teams
- Admissions to and number of days in crisis houses
- Detention in hospital under mental health law
- Adverse events (e.g. complications, mortality)

#### Search strategy

Eligible studies will be identified using the following search terms (the example provided applies to the MEDLINE database, with ‘ti,ab’ meaning title and abstract, ‘sh’ meaning subject heading and ‘pt’ meaning publication type):

i. Mental health nurs$.ti,ab. OR psychiatric nursing.sh. OR psychiatric nurs$.ti,ab.
ii. Severe mental illness.ti,ab. OR mental disorder.sh. OR mental illness.ti,ab. OR schizo$.ti,ab. OR schizophrenia.sh. OR bipolar disorder.sh. OR psychos?s.ti,ab. OR psychotic.ti,ab. OR psychotic disorders.sh. OR psychotic affective disorders.sh. OR disorders with psychotic features.ti,ab. OR psychotic depression.ti,ab.
iii. Patient admission.sh. OR patient readmission.sh. OR hospital admission.ti,ab. OR hospital readmission.ti,ab. OR unplanned readmission.ti,ab. OR hospitalization.sh. OR readmission rate.ti,ab. OR length of stay.sh. OR emergency department presentation.ti,ab. OR admission to home treatment.ti,ab. OR access to crisis intervention.ti,ab. OR drop-in treatment.ti,ab. OR drop-in care.ti,ab. OR drop-in unit.ti,ab. OR drop-in centre.ti,ab. OR home intervention.ti,ab. OR home therapy.ti,ab. OR home care.ti,ab. OR home management.ti,ab.
iv. Observational study.pt. OR cross-sectional study.pt. OR cohort study.pt. OR longitudinal study.pt. OR epidemiologic study.pt. OR case-control study.pt. OR controlled clinical trial.pt. OR randomized controlled trial.pt. OR non-randomised controlled trial.pt. OR quasi-experimental study.pt. OR clinical trial.pt. OR comparative study.pt.

The search will only include articles published in the English language. No restrictions on the date of publication will be applied to the searches.

The search will be conducted using the following electronic databases, from their inception:

- EMBASE [OVID]
- CINAHL [EbscoHost]
- Health Source: Nursing/Academic Edition [EbscoHost]
- MEDLINE [OVID]
- Nursing & Allied Health Database [ProQuest]
- Ovid Nursing [Ovid]
- ProQuest Dissertations and Theses Global [ProQuest]
- PsychINFO [Ovid]
- PubMed [NCBI]
- The Cochrane Library
- Web of Science [Thomson Reuters]

Also, the reference lists of included studies will be hand-searched to identify potentially eligible studies. Trial registers also will be searched to identify on-going or unpublished trials, including http://www.ClinicalTrials.gov, http://www.who.int/trialsearch/ and http://www.anzctr.org.au/.

#### Study selection

Records identified in the searches will be exported to reference management software (Endnote). Duplicate records will be reviewed, and if appropriate, excluded. The reference management file will be exported to Covidence (a web-based software platform for systematic reviews) for screening. Titles and abstracts of all identified articles will be screened for eligibility against the review selection criteria. Two reviewers (KM, AJ) will conduct the screening process, independently. Discussions will occur with a third reviewer if disagreements occur (RG). The same process will be followed at the full-text screening stage of the review. A PRISMA (Preferred Reporting Items for Systematic Reviews and Meta-analyses) flow chart will be compiled to summarise the study selection process.

#### Data extraction

Data from eligible studies will be extracted using a data extraction tool developed exclusively for this review. The Covidence platform will be utilised to administer the study. The tool will collect information on several different parameters. This will include study/report characteristics (i.e. author, year of publication, country), research methodology (i.e. study design, number of study centers), participants (i.e. sample size, sampling frame, selection criteria, setting, demographic and clinical characteristics of participants), interventions (i.e. detailed description of intervention[s], level of exposure), comparators (i.e. detailed description of comparator[s], level of exposure), outcomes (i.e. primary and secondary outcomes, outcome measures), results (i.e. findings reported against each outcome) and new references (i.e. identification of potentially eligible studies). Data extraction will be performed by two reviewers (KM, AJ), independently, with disagreements resolved by discussion with a third reviewer (ML).

#### Risk of bias

The risk of bias of observational studies will be assessed using the Risk Of Bias In Non-randomised Studies - of Interventions (ROBINS-I) tool [16]; this will be administered via the Covidence platform. The ROBINS-I tool evaluates the risk of bias across seven distinct domains, including baseline and time-varying confounding, participant selection, intervention classification, co-intervention, missing data, outcome measurement and selective reporting bias. Two reviewers (MJ, ML) will independently evaluate the risk of bias and rate studies as having low, moderate, severe, critical or unclear risk of bias. If a disagreement occurs between reviewers, a third reviewer will be consulted (DB).

The risk of bias of clinical trials will be evaluated using the Cochrane Collaboration risk of bias tool [17]. The Cochrane tool assesses risk across seven domains, including sequence generation, allocation concealment, blinding of participants, personnel and outcome assessors, incomplete outcome data, selective outcome reporting and other sources of bias. Two reviewers (MJ, ML) will independently assign a judgement related to the risk of bias for each item, with a judgement of ‘Yes’ indicating low risk of bias, ‘No’ indicating a high risk of bias and ‘Unclear’ indicating unclear or unknown risk of bias. If consensus cannot be achieved between the two reviewers, a third reviewer will arbitrate (DB).

#### Data synthesis

Where there are three or more studies that report similar designs, interventions and outcome measures, data will be combined by way of meta-analysis to calculate pooled effect estimates and their 95% confidence intervals using a random-effects model. This analysis will be conducted using Review Manager software (RevMan, version 5.3, Copenhagen, The Nordic Cochrane Centre, The Cochrane Collaboration, 2017). In the event of substantial statistical (*I*^2^ ≥ 50%), clinical or methodological heterogeneity, or where there are too few studies, results will be presented using a systematic narrative synthesis. Two reviewers (ML/MJ) will undertake the synthesis. Disagreement between reviewers will be resolved via discussion with a third reviewer (RG).

Subgroup analyses will be performed to explore the influence of nurses’ clinical practice setting (community, outpatient, inpatient, country), highest mental health qualification (diploma, master’s, PhD), psychosocial intervention training (e.g. cognitive behavioural therapy) and years of mental health nursing experience (< 5 years, 6–10 years, > 10 years). Similarly, sensitivity analyses will be undertaken to determine whether the outcomes of the study are affected by risk of bias (i.e. excluding from the analysis any studies judged as having severe/critical/unclear risk of bias).

#### Risk of bias across studies

Where there are at least ten studies included in a meta-analysis, funnel plot asymmetry will be tested in order to assess the presence of publication bias and small study effects. The Grading of Recommendations Assessment, Development and Evaluation (GRADE) approach will be used to judge the quality of evidence for all outcomes [18]. This approach rates the quality of the body of evidence by considering the following aspects: study design, risk of bias, imprecision, inconsistency, indirectness and magnitude or effect. The quality of the evidence will be rated as either high, moderate, low or very low.

## Discussion

Relapse is common in people with SMI. The implications of relapse are considerable, impacting not only patients and their carers but also the mental health workforce and policy makers. Prevention of relapse or maintaining an optimum level of well-being is an important objective of mental health care and a core skill of mental health nurses.

It is acknowledged that relapse in people with SMI can be difficult to measure. Some people with SMI are admitted to hospital for reasons other than relapse, such as the initiation of clozapine treatment. There are also instances when people with SMI relapse that do not go into hospital (Schennach et al. [8]); in such cases, the person may be cared for at home by a crisis resolution or home treatment team. Nonetheless, given that the majority of cases of relapse in SMI result in hospital admission (Schennach et al. [8]) and hospitalisation is associated with considerable psychosocial and economic burden (Ascher-Svanum et al. [10]; Gerson & Rose [11]), admission to hospital is considered a suitable proxy measure of relapse; accordingly, hospital admission is likely to be an important indicator of the effectiveness of mental health nursing in preventing people with SMI from experiencing a relapse.

Mental health nurses arguably play an important function in building relationships, providing social support (such as help with housing, access to employment and education opportunities) and facilitating the safe administration of medications (Department of Health [15]). However, on a hierarchy of MHN clinical outcomes, preventing relapse should be a key therapeutic aim of SMI (Olivares et al. [12]); it is also perhaps the most significant and most meaningful outcome for patients, particularly when the consequences of relapse are taken into consideration. Relapse is also costly, and therefore, it should be an important consideration for policy makers in any future health workforce planning.

This review is timely for mental health nursing, with recent findings from a large multi-national observational study demonstrating the impact of nurse educational qualifications on patient mortality; specifically, the association between the provision of care by bachelor degree qualified nurses and increased probability of survival in a general hospital setting (Aitken et al. [19]). Of course, this work only examined medical-surgical nurses and further work needs to be done to determine whether a qualification in mental health nursing impacts health outcomes in patients with SMI (recognising that MHN training programs vary across countries and any future work in this area will need to take into consideration differences in the educational preparation of MHNs). As a first step in addressing this knowledge gap, this review will explore the clinical potential of mental health nursing in preventing relapse in individuals with SMI. The findings will help to inform future policy, research and education in this area; it may also assist decision makers with future mental health workforce development and planning.

## Abbreviations

ED: Emergency department
MHN: Mental health nurse
ROBINS-I: Risk Of Bias In Non-randomised Studies - of Interventions
SMI: Severe mental illness
